# The Temporary Relief of Turbinal Distension

**Published:** 1907-11-02

**Authors:** F. P. Atkinson


### EDITOR'S LETTER-BOX.

[Our Correspondents are reminded that prolixity is a great bar to publication, and that brevity of style and conciseness of statement greatly facilitate early insertion.]

### THE TEMPORARY RELIEF OF TURBINAL DISTENSION.

Sir,—There are few things more worrying than cases of turbinal atony and distension. Where the block is very severe nothing will afford relief but the application of the galvanic cautery; but in slight cases a great deal of relief is obtained from the use of cocaine and menthol snuff—1 grain of the former, 2 grains of the latter, mixed up with 100 grains of boric acid, or a spray consisting of 10 grains of cocaine, 20 to 30 menthol, 1 drachm of oil of almonds, and 1 oz. of liquid vaseline; but I have found from personal experience that the vapour given off from menthol crystals by itself often gives as much relief as either the compound snuff or spray—and where a solution of cocaine is used by itself the crystal vapour afterwards comes in as a very useful adjunct. The point mentioned is a very small one, but I think it may prove useful.

I am, sir, yours faithfully,

F. P. Atkinson, M.D., C.M.

